# Chemosensitive Thin Films Active to Ammonia Vapours

**DOI:** 10.3390/s21092948

**Published:** 2021-04-22

**Authors:** Agnieszka Brochocka, Aleksandra Nowak, Hanna Zajączkowska, Marta Sieradzka

**Affiliations:** 1 Department of Personal Protective Equipment, Central Institute for Labour Protection—National Research Institute, 90-133 Lodz, Poland; alnow@ciop.lodz.pl (A.N.); hazaj@ciop.lodz.pl (H.Z.); 2 Faculty of Materials, Civil and Environmental Engineering, University of Bielsko-Biala, 43-309 Bielsko-Biala, Poland; msieradzka@ath.bielsko.pl

**Keywords:** sensor, signal processing, chemical sensor, personal protective equipment, gas detection, ammonia, spraying, drop-casting

## Abstract

The paper presents various dispersive systems developed for sensing toxic substance—ammonia. Polycarbonate dissolved in methylene chloride was used as a polymer matrix, which was enriched with: multi-walled carbon nanotubes (MWCNs), reduced graphene oxide (rGO) and conductive polymer (polyaniline—PANi). Dispersive systems were applied to the prefabricated substrates with comb electrodes by two methods: spraying and drop-casting, forming an active chemosensitive to ammonia vapours films. The spraying method involved applying the dispersion to the substrate by an aerograph for a specific time, whereas drop-casting involves depositing of the produced dispersive systems using a precision automatic pipette. The electrical responses of the obtained films were examined for nominal concentrations of ammonia vapours. Different types of dispersions with various composition were tested, the relationships between individual compounds and ammonia were analysed and the most promising dispersions were selected. Sensor containing rGO deposited by drop-casting revealed the highest change in the resistance (14.21%).

## 1. Introduction

The presence of volatile chemicals in the working environment often contributes to the loss of life and health in humans. The inhalation of chemicals causes various defensive reactions of the body, such as drowsiness, fatigue and irritation of the mucous membranes of the nose, throat and eyes. Severe effects of long-term exposure to such chemicals in concentrations exceeding the maximum admissible concentrations include headache, nausea, diarrhoea, intoxication or even death. The previously developed selective sensors of volatile chemicals do not include the solutions that enable the detection of substances which would be suitable for implementation in the respiratory protective devices.

The presence of harmful chemicals is one of the factors related to the pollution at workplaces [1]. The air pollution, concentration of harmful and hazardous substances, as well as frequency and duration of the exposure can affect several aspects of human health and well-being [2]. Air pollution is defined as a complex mixture of particles in the air, biological ingredients and gases, which puts humans, plants and animals at risk. To prevent and control the exposure for volatile compounds the evaluation of the basic risk factors is necessary, as well as use of personal protective equipment and assessment of its service life [3]. According to the statistical estimations, about 3,000,000 people lose their lives every year because of air pollution and the related ailments [4]. Epidemiological data indicate the harmful effects of air pollution on human health [5]. Depending on the nature of human exposure to harmful and hazardous substances, various effects of air pollution have been observed. The most common ones include the deteriorated activity of the immune system and cardiovascular problems [6], breathing problems and lung related diseases [7] but also skin irritation [8]. The two main routes of human exposure to air pollution include inhalation and skin contact. As far as occupational exposure to chemicals is concerned, the contribution of other routes such as ingestion seems to be limited and tends to occur only incidentally [9]. That is why substance penetration through the respiratory tract is regarded as a serious problem [10], and the best protective measures and equipment should be used [11].

The respiratory tract can be protected by using filtrating equipment, which cleans air and provide it free of pollutants. Considering the fact that the processes of toxic substances capturing may vary depending on the form of the substances (vapours, gases, solid and liquid particles), different filtering and absorbing materials are used. From the user point of view, it is important that the absorber had an indicator which would actively and clearly inform the operator when the useful life of the sorption filter, responsible for the absorption of vapours and gases of dangerous substances, is about to expire [12]. The problem can be solved by introducing a sensor into the absorber structure, enabling the determination of the absorber protective action by measuring the substance concentration behind the sorption filter.

The principle of the operation of the sensor is based on the changes in the physical and chemical characteristics of the sensory material as a result of absorption or desorption of the vapours of chemicals present in its surrounding environment. That is why materials with the well-developed specific surface are used in the sensor designs. They are typically and commonly used in such products as carbon sorbents, structural and insulating carbon-based porous materials, carbon fibres and graphite greases [12]. The authors of some papers [13,14] demonstrated the reaction of sensors based on single-walled carbon nanotubes (SWCNs) for ammonia vapours (ca. 1 ppm) and sulphur dioxide. Detailed tests related to the reaction time, sensitivity and reproducibility of their operation time were carried out. The tests revealed excellent reproducibility of the sensor operation with a complete and quick signal recovery time [13]. Deeper studies on the sensing mechanism of SWCNs based sensor was also carried out [14]. Another paper [15] demonstrated that structural features greatly affect the characteristics of carbon nanotubes. The more developed the specific surface, the greater the absorption of vapours and gases present in the air, which is related to a higher sensitivity of sensors based on carbon nanotubes and allow to detect harmful and hazardous substances. The sensitivity of carbon nanotubes to such substances as hydrogen sulphide, acetone, nitrogen dioxide, ammonia, toluene and benzene has also been proven [16], which makes it possible to use them as active indicators of the protective time of respiratory protective equipment [17].

Graphene is another material of interest in the literature because of its unique mechanical properties, like high heat and electrical conductivity as well as a large specific surface [18]; the same applies to graphene derivatives such as graphene oxide (GO) and reduced graphene oxide (rGO). Owing to the excellent chemical and physical stability of GO, the authors of paper [19] used GO to produce sensors based on SiO_2_ nanotubes to detect ethanol vapours. Tests were carried out for the concentration range of 50–500 ppm.

The further part of this paper describes the methodology of deposition of thin films from the prepared dispersive systems, and the sensory properties of the obtained films. The available literature applies mainly to the physical and chemical characteristics of the materials used in various types of sensors, their qualitative composition and impact on chemicals. Still, there is no information concerning the application of chemosensitive films to different substrates, which can be the manufacturers’ “know-how” in many cases. The study was aimed to produce dispersive systems in the polymer matrix and deposit them using two methods: spraying and drop-casting in order to achieve the smallest possible material losses and to obtain the best possible chemosensitive films active to ammonia vapours.

## 2. Materials and Methods

The idea described in this paper is about the development of the fabrication method of sensitive sensors for volatile hazardous vapours. In the literature a lot of sensitive sensors were already presented, but often high performance of the devices goes with difficult and multistep fabrication process [20] including long and usually problematic synthesis route of the active materials [21], which are an opposite of energy, money and time saving solutions. In this approach simple method of dispersion preparation (with use of commercially available active materials) and single-step fabrication process of sensors are presented. This path will allow the commercialisation of sensors fabrication and use them in the respiratory protective equipment, as cheap, sensitive and reversible sensors.

### 2.1. Materials

In order to fabricate chemosensitive thin film sensors active on toxic and volatile organic compounds (VOCs) such as ammonia vapours (NH_3_) innovative multicomponent dispersive systems composed of methylene chloride (CH_2_Cl_2_, STANLAB, Lublin, Poland) and polycarbonate (PC, LEXAN, General Electric Company, Boston, MA, USA) as polymer matrix and polyaniline (PANi, Sigma Aldrich, St. Louis, MO, USA), multi-walled carbon nanotubes (MWCNs, Ossila, Sheffield, UK) and reduced graphene oxide (rGO, University of Bielsko-Biala, Bielsko-Biala, Poland) as active materials (additives) have been used. All materials were used as received without any purification or another preparation. In order to define impact of each component on sensing properties a series of samples with different combination of additives were fabricated. The qualitative and quantitative compositions of each version of dispersion are summarised in Table 1.

### 2.2. Research Methods

#### 2.2.1. Additives Characterization: BET and SEM Measurements

The specific surface area (SSA) measurements of pristine additives (MWCNs, rGO and PANi), were performed by the Quantachrome^®^ ASiQwinTM -Automated Gas Sorption Data analyser (Quantachrome Ins, Boynton Beach, FL, USA). The SSA values were calculated using the BET (Brunauer–Emmett–Teller) method. Before the measurement, samples were dried in a vacuum in a desorption station (with outgassing temperature: 150 °C). Liquid nitrogen at temperature of −195.9 °C was used during the measurements.

The images of scanning electron microscopy (SEM) were obtained by field emission scanning electron microscope—SU8010 HITACHI, Chiyoda, Tokyo, Japan.

#### 2.2.2. Preparation of Dispersive Systems

The dispersive systems preparation method was based on two stages of mixing: mechanical, with magnetic stirrer and using ultrasounds. Mechanical mixing was used to evenly distribute all components in the polymer matrix whereas ultrasounds were used to increase homogeneous of the dispersion and to avoid aggregation of additives. At the first stage, the appropriately weighed additives were mixed with the polymer matrix and were tightly closed in glass vials with magnetic stirrers and mechanically mixed at 150 rpm for 24 h. Then all the prepared dispersive systems were exposed to ultrasounds for 8 h using 250 W sonicator. During sonication process, the temperature of the dispersions was kept below the boiling point temperature of used solvent (T_bp_ = 39.6 °C) by use of water/ice bath.

#### 2.2.3. Thin Films Deposition Methods

In this paper two deposition methods—spraying and drop-casting—were compared. In order to ensure coherent results, the process of film deposition was carried out under identical environment conditions, with temperature and humidity control.

∙ spraying

Pressure gauge was used to supply the correct pressure (1.0 bar) of the dried and compressed air from the network to the aerograph (AD-7734 Adler, Lodz, Poland). Then the aerograph tank was filled with the adequate version of the previously prepared dispersive system and sprayed onto the substrate with comb electrodes for a strictly determined and controlled time—4 s. The dispersive systems were sprayed perpendicularly to the substrate from the distance (height) of 8 cm.

∙ drop-casting

The developed dispersive systems were dispensed from an automatic precision pipette perpendicularly to the substrate. The inner diameter of the automatic pipette was 0.11 mm, and its total volume was 0.2 mL.

After the deposition of chemosensitive films the samples were dried in a vacuum dryer (BINDER GmbH, Tuttlingen, Germany) for 2 h at 80 °C to remove residue of the moisture.

#### 2.2.4. Electrical Response Examination of the Chemosensitive Films

The test rig was equipped with three rotameters, which were responsible for supplying the right quantity of compressed and dried air to the measurement system. Gas detectors—Dräger X-am 7000 analysers (Drägerwerk AG & Co. KGaA, Lübeck, Germany) were used to measure the concentration of the ammonia vapours in the control and measurement chambers. Aerosol was produced in the evaporator, where the test substance was mixed with compressed and dried air. The rotameters were used to achieve the desired concentration of the test substance, at the nominal concentration equal to the maximum admissible concentration (MAC) established by European Union Commission Directive, at value of 20 ppm (± 3 ppm) [22]. The test was carried out at the volumetric airflow of 30 L/min.

Before the test cycle, the electrodes were connected to a digital laboratory multimeter (KEYSIGHT Technologies 34461A Digit Multimeter, Truevolt, Santa Rosa, CA, USA), which was used to record the change of the chemosensitive layer resistance in time.

The measurements revealed which versions of the prepared dispersive systems were characterized by good sensitivity to the applied ammonia vapours. The main criterion for sensitive sensor was to obtain high percentage resistance change (S) during exposure for the ammonia vapours. The value for this parameter was determined using the equation:

S = ((R_MAX_ − R_0_)/R_0_) × 100%, (1)

where: S is the percentage change in the resistance value, R_MAX_ is the maximum change value of the resistance after the sensor reaction with ammonia vapours, R_0_ is the original resistance value (after exposing the test sensor to clean air) [21].

## 3. Results and Discussion

### 3.1. Additives Characterisation

It is known that gas diffusion occurs more easily in porous structures [23], the reaction between gas molecules and the porous-thin film is more efficient when surface-to-volume ratio is as high as possible [24].

The surface area analyses of MWCNs, rGO and PANi are shown in Table 2. The presented data shows that the MWCNs and rGO have similar SSA values, while the PANi specific surface area is seven times lower. Moreover, all of the samples contain in their structure mesopores with the average values of 2.96 nm, 2.95 nm and 3.29 nm for MWCNs, rGO and PANi, respectively. The lowest pore volume was determined for PANi, which also has the lowest SSA. This might mean that this material possesses the smallest number of open-type pores while the highest SSA and pore volume was determined for rGO, which may suggest the best sorption properties of this material (most active sites for the adsorption).

**Figure 1:**
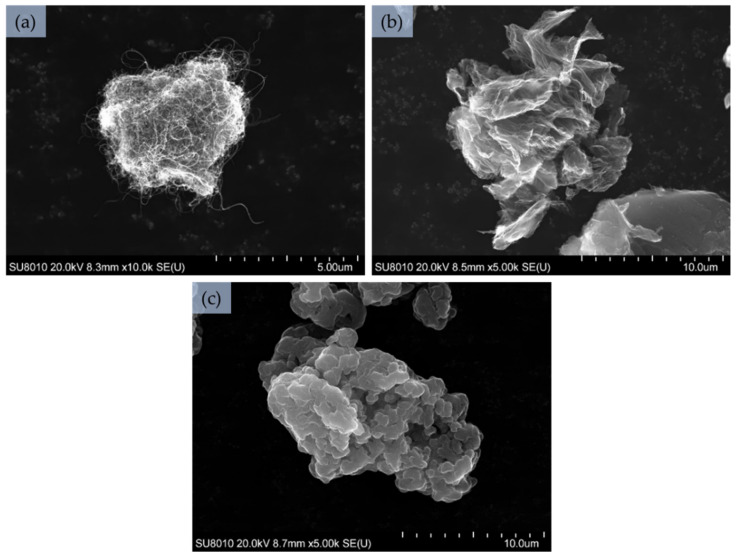
SEM images of pristine additives: (**a**) MWCNs, (**b**) rGO, (**c**) PANi.

The SEM images of pristine MWCNs, rGO and PANi are shown in Figure 1. Figure 1a presents aggregate of MWCNs, which creates round objects with average diameter of 5 µm, consisting of long and curled structures with average diameter of 12 nm. The MWCNs aggregates create a larger number of holes between the structures which indicate a large number of pores and potential sites for adsorption of test substances, which is confirmed by high BET SSA and can contribute to the short response time, reversibility and high sensitivity of the sensor. Figure 1b shows rGO aggregate with the more irregular shape and diameter from 11 to 15 µm, consisting of crumpled paper-like structures, with folded edges which makes the assessment of the thickness of single structures very difficult. These kinds of aggregates, similar to MWCNs, create a large number of empty spaces (confirmed by the highest BET SSA) which can be a potential adsorption sites and influence on the good sensing properties. PANi also creates aggregates with the irregular shape and size from 9 to 14 µm, shown in the Figure 1c. These aggregates consist of permanently connected with each other smooth particles with diameter of 1 µm. It is clear, that such structures create only a small number of pores and potential adsorption sites, which is confirmed with the lowest BET SSA and pore volume, in comparison to MWCNs and rGO.

### 3.2. Morphology of Thin Films

#### 3.2.1. Assessment of the Morphology of Chemosensitive Films Deposited by Spraying

**Figure 2:**
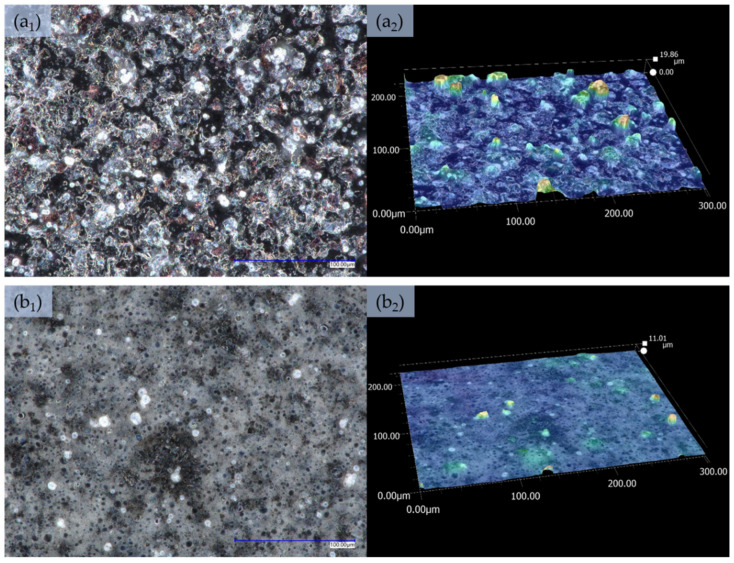
Images from an optical microscope presenting the morphology of films (mag ×1000) produced by spraying, and the depth profiles presenting the roughness of the layers (mag ×1000): (**a_1_**,**a_2_**)—D 4 dispersion, (**b_1_**,**b_2_**)—D 7 dispersion.

Figure 2 and Figure S1 (in Supplementary Materials) present an optical microscope images and depth profile images obtained using Keyence VHX-7000 microscope for all investigated dispersion deposited by spraying method. As can be notice, the surface morphology of all samples which contain PANi in the dispersion is similar (D 1—Figure S1(a_1_), D 2—Figure S1(b_1_), D 3—Figure S1(c_1_), D 4—Figure 2(a_1_)). Observed structures create continuous and homogeneous morphology which suggest good dispersion of the additives in the polymer matrix. The main difference in the morphology of these four samples is the roughness which is equal to: 14 µm for D 1 (PANi—Figure S1(a_2_)), 25 µm for D 2 (PANi + MWCNs—Figure S1(b_2_)), 35 µm for D 3 (PANi + rGO—Figure S1(c_2_)) and 20 µm for D 4 (PANi + MWCNs + rGO—Figure 2(a_2_)). These measurements indicate that the quantity of other additives (two allotropic forms of carbon—MWCNs and rGO) is too low to significantly affect the morphology of thin films deposited with an aerograph but have an impact on the roughness. High roughness may suggest some form of the aggregation of carbon additives in the dispersion. Figure S1(d_1_,d_2_) shows the morphology and depth profile of the film developed from D 5 dispersion which contains only MWCNs in the polymer matrix and reveal differences in the morphology in comparison to thin films containing PANi, as well as lower roughness (equal to 12 µm). On the film surface, there are visible multiple spherical objects, which (as the depth profile suggests) can be assigned to the voids left after air bubbles (from spraying process). When film is applied by spraying with an aerograph, the solution is aerated creating aerosol in order to achieve a uniform and homogeneous film. The microscopic images of the film based on the D 6 dispersive system, contains only rGO in polymer matrix (Figure S1(e_1_,e_2_)) also show noticeable spherical structures, which originate from air bubbles, (similar to the film based on D 5 dispersion) and even lower roughness of 6 µm. The morphology of a thin film (Figure 2(b_1_,b_2_)), which contains both MWCNs and rGO (D 6), is characterised by structures similar to the films containing individual components and roughness of 11 µm.

#### 3.2.2. Assessment of the Morphology of Chemosensitive Films Deposited by Drop-Casting

**Figure 3:**
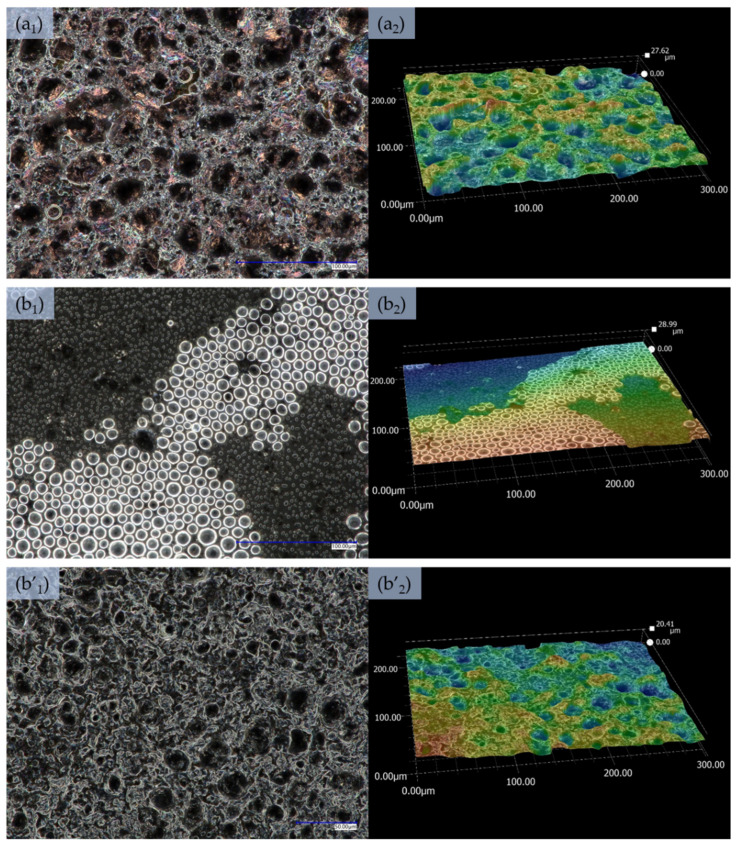
Images from an optical microscope presenting the morphology of films (mag ×1000) produced by drop-casting, and the depth profile presenting the roughness of the layers (mag ×1000): (**a_1_**,**a_2_**)—D 4 dispersion, (**b_1_**,**b_2_**,**b’_1_**,**b’_2_**)—D 7 dispersion in two different places.

Figure 3 and Figure S2 (in Supplementary Materials) present an optical microscope images and depth profile images for all investigated dispersion deposited by drop-casted method. Similar to the sprayed films, the morphology of drop-casted dispersions containing PANi (D 1—Figure S2(a_1_), D 2—Figure S2(b_1_), D 3—Figure S2(c_1_), D 4—Figure 3(a_1_)) did not vary from each other visibly. The morphology of these samples characterised with larger and more densely packed structures in comparison to sprayed films (Figure 2a, Figure S1a,b,c). This suggest that independently from the used deposition method, morphology is strongly controlled by PANi. The depth profiles clearly show that the visible structures are holes/pits in the film (with roughness of 16 µm for D 1—Figure S2(a_2_), 24 µm for D 2—Figure S2(b_2_), 20 µm for D 3—Figure S2(c_2_) and 28 µm for D 4—Figure 3(a_2_)), which can be attributed to free organisation and aggregation of the materials during the solvent evaporation from the applied film.

The morphology of the film which contains only MWCNs (D 5—Figure S2(d_1_,d_2_)) in a polymer matrix is characterised by larger and more rounded structures, which, similar to the other films developed by drop-casting, form pits in the applied dispersion causing roughness of 28 µm. The film morphology is a result of specific aggregation of nanoparticles in the polymer matrix. Contrary to the sample with MWCNs (D 5), D 6 with rGO do not exhibit major morphology defects as holes or pits in the film (Figure S2(e_1_,e_2_)). Perfectly round, densely packed structures which slightly protrude above the film surface are visible. The rGO can be either aggregating or getting organised to form the above-mentioned structures in the presence of a polymer matrix. Film deposited from D 7 dispersion (MWCNs and rGO) characterised with two types of structures, identical to those observed for the films developed from individual components: deep holes (Figure 3(b’_1_,b’_2_)) and perfectly round flat structures (Figure 3(b_1_,b_2_)), leading to film roughness of 20–29 µm.

It was determined that alike the samples deposited by spraying, the drop-casted films based on the dispersive systems containing PANi, are characterised by highly similar morphology. However, films deposited by both methods from dispersive systems containing MWCNs and rGO without PANi, show completely different structures. Thus, it was revealed that the introduction of other additives to the dispersion from PANi did not significantly change the morphology of the film.

### 3.3. Sensors’ Electric Response to Ammonia Vapours

Sensors based on the developed dispersive systems were examined for their reaction to the vapours of harmful and hazardous substance—ammonia, at the nominal concentration equal to 20 ppm—MAC [22]. The tests were carried out in a glass chamber, provided with an efficient ventilation system. Table 3 summarises the obtained values of the resistance change (S) for the developed sensors.

**Figure 4:**
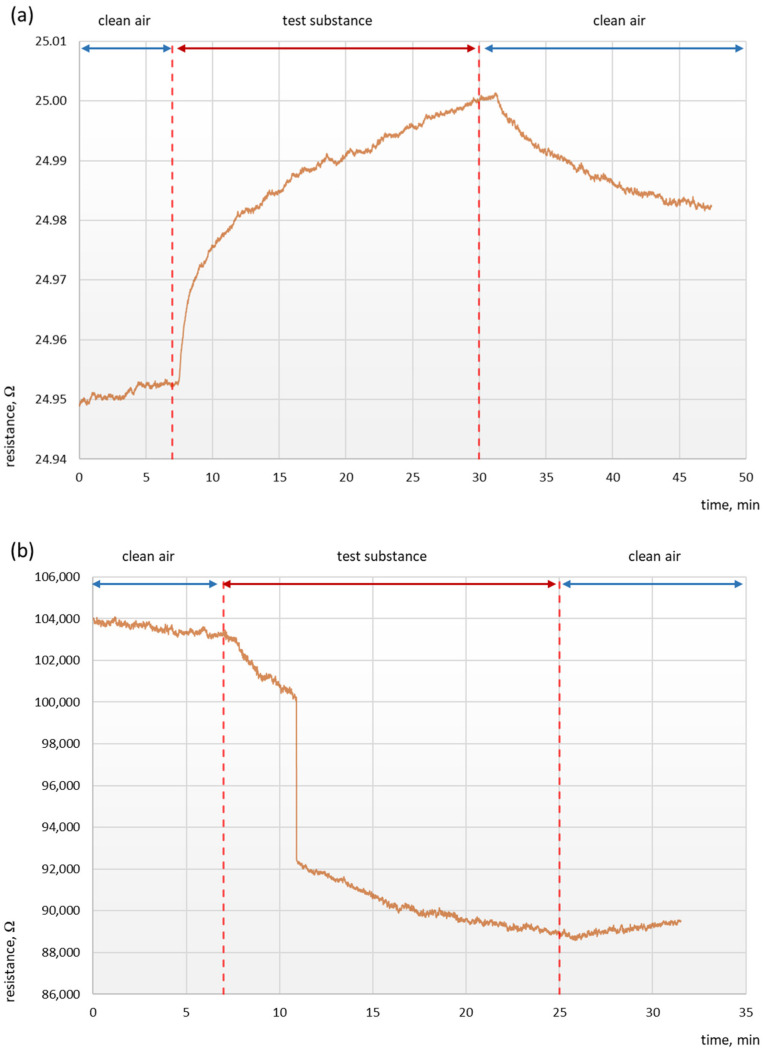
The resistance response of the sensors deposited from: (**a**) D 5 dispersion by spraying and (**b**) D 6 by drop-casting.

Films from D 1 and D 3 dispersions deposited by both methods and D 6 dispersion deposited by spraying revealed resistances above the multimeter scale (above 10 GΩ)—there was no layer conductivity required to conduct the measurement. The resistance response in time for all working devices are presented in Figure 4 and Figure S3 (in Supplementary Materials). The highest resistance change (S)—14.21% was recorded for drop-casted dispersion D 6 (containing rGO in polymer matrix)—Figure 4b. It is well known that the more developed the surface is, the greater the absorption of vapours and gases occurs. The presence of the perfectly round, densely packed structures visible in the microspore image of film deposited from D 6 dispersion by drop-casting (Figure S2(e_1_)), and the highest SSA and pore volume lead to the high sorptive properties which are one of the reasons of high sensor sensitivity. The rest of the working sensors revealed S not higher than 0.62% regardless of used deposition technique. Obtained low values of S do not deviate from the literature values for similar thin films sensors exposed for 20 ppm of ammonia vapours. RGO based sensors, which in this research characterised with the highest resistance change (14.21%) in the literature obtained S = 3.32% after exposure for 10 ppm of ammonia vapours [21] and 22% after exposure for 100 ppm of NH_3_ [25]. PANI based sensors were also deeply investigated concerning nanostructures of PANI, drop-casted conventional PANI exhibited 1.47% S and drop-casted nanostructured PANI reached 4.37% S after exposure for 92 ppm of NH_3_ [26]. While PANI and MWCNs based sensor deposited on fabric obtained even 90.70% sensitivity for 100 ppm of ammonia vapours (it is worth to mention that in this case special multi-step deposition procedure was used), in the same paper only PANI based sensor and only MWCNs based sensor were also characterized and achieve S value of 23.46% and 6.92%, respectively [20]. It was noticed that in the literature measurements of sensor reaction are conducted under enormous range of ammonia vapours exposure, reaching even 6400 ppm [23], resulting in increased of sensors sensitivity for such high concentrations of NH_3_.

Despite the low value of S, equally important is the time response of the sensors to changes in concentration as well as to disappearance of the analyte. Therefore, the main key of working and reusable sensors is the reversion of the resistance to its nominal value when it is not exposed to the analyte. Such behaviour was observed for films deposited from dispersions D 2 (Figure S3a), D 4 (Figure S3c), D 5 (Figure 4a), D 7 (Figure S3f) by spraying and D 2 (Figure S3b), D 4 (Figure S3d) and D 6 (Figure 4b) by drop-casting. The most visible and sharp response of the sensor was observed for D 5 dispersion (Figure 4a) and D 7 dispersion (Figure S3f), both deposited by spraying, where at the beginning of the experiment during exposure for the clean air the resistance value stayed constant. Subsequently after the introduction of the ammonia vapours to the measurement chamber immediate response was recorded. The resistance increased gradually (D 5) or decreased reaching plateau (D 7), until closing the valve supplying ammonia vapours and again starts to return to the nominal value after exposure of the sensor for clean air. The change of the resistance at the second clean air introduction, occurred after some time, because the chamber had to be fill out again with air and get rid of the NH_3_ residuals.

Attention should be paid also to the type of the resistance response. In the sensors fabricated from dispersions containing MWCNs and PANi (D 2, D 4, D 5), the resistance increased during the ammonia vapours exposure (film conductivity decreased), while in the sensor fabricated from dispersion containing only rGO (D 6) and rGO and MWCNs (much lower S) (D7), the resistance decreased under the influence of ammonia vapours (film conductivity increased). These results came from interactions between components of dispersions and ammonia which is a strong reducing and electron donor material. The increase of the resistance for sensors containing MWCNs and PANi (which are p-type semiconductors) is probably the effect of NH_3_ chemisorption, causing charge transfer between ammonia and MWCNs (reducing hole density) [23] and PANi (leading to the localization of polarons influencing the mobility) [27], bringing to the decrease of the amount of charge carriers and lowering the conductivity in both materials. In contrary rGO as an electron donor with a large number of functional groups and defects creates a lot of sites for chemisorption of ammonia. This leads to charge transfer between NH_3_ and rGO and increase of the charge carriers causing decrease of resistance—higher conductivity [21]. These opposite effects caused low final sensitivity of the dispersions, which contain both allotropic forms of carbon. It can be attributed to the fact that the responses of the additives balance out resulting poor S.

Another important factor is the average conductivity of the layer, it was noticed that sensors deposited by spraying revealed lower conductivity (higher resistance) then sensors deposited by drop-casting for each dispersive system. However, the reasons of this behaviour must still be examined.

The key factors that contribute to the improvement in the sensors detection efficiency and sensitivity are related to the conductivity of the material/blend and the amount of sites for adsorption, which are related with active surface area and morphology and can be tuned by: composition, preparation and deposition method of the dispersion. Attention shall also be paid to the fact that the mechanism of toxic and hazardous substance detection is based on the principle of the sensor chemical resistance change and its physical and chemical interaction with the detected substance, as well as on the thickness of the layer which is the active substrate that reacts to harmful and hazardous substances.

## 4. Summary

Sensor devices, based on thin films, active to ammonia vapours were deposited to the prefabricated substrates with comb electrodes using two methods: spraying and drop-casting. They were exposed to ammonia vapours, whose concentration in a mixture with clean air amounted to 20 ppm. This concentration is not a sensitivity limited value for developed in this paper sensors, it was chosen according to MAC established by European Union. Further studies are needed to define the limitation of the devices. The developed sensors were examined using an optical microscope, which helped to assess the differences in the morphology and the roughness of developed sensory films, the most complex morphology was observed for dispersion with rGO deposited by drop-casting. The resistance analysis allowed to characterize the fabricated sensors in terms of sensor sensitivity value (S), reversion of the process and quality of obtained results. The measurements show that the sensor ensures good sensitivity (D 6—14.21%) and satisfactory activity (quick response) and reversibility of response to the vapours used at a low concentration of ammonia (20 ppm). Before these measurements, components of the dispersive systems were characterized by the particles and pore sizes and specific surface area. In this case, rGO exhibited the highest SSA and pore volume, which suggested the best sorptive properties what was confirmed by the highest S obtained for sensor based on rGO. Dispersions with various composition were analysed and the most promising dispersion—containing rGO deposited by drop-casting was obtained. The sensor model developed in this paper can be potentially extended by a change in the quantitative composition, but also by measurements in different environmental conditions including various temperature and humidity and wide range of NH_3_ concentration, which opens the possibility of further improvement in the sensor quality. Nonetheless, it can be a promising material to be used in a portable, low cost and easy fabricated sensor reacting to ammonia vapours at room temperature. Both the spraying and drop-casting methods are acceptable methods of applying thin chemosensitive films, active to ammonia vapours.

## Figures and Tables

**Table 1:** Composition of the developed dispersive systems.

Identification of the Dispersive System	Polymer Matrix		Additives		
	Methylene Chloride, mL	Polycarbonate, g	MWCNs, mg	rGO, mg	PANi, mg
D 1	70	1	-	-	1000
D 2			50	-	
D 3			-	50	
D 4			50	50	
D 5			50	-	-
D 6			-	50	
D 7			50	50	

**Table 2:** Specific surface area (BET), pore size and pore volume of PANi, MWCNs and rGO.

Sample	Surface Area (BET), m^2^/g	Pore Size, Nm	Pore Volume, cm^3^/g
MWCNs	349.69	2.96	0.34
rGO	416.13	2.95	0.37
PANi	53.96	3.29	0.07

**Table 3:** Summary of the resistance change (S) value (in %) of the sensitive films.

Dispersion Type	Resistance Change S, %	
	Film Deposited by Spraying	Film Deposited by Drop-Casting
D 1	-	-
D 2	0.62	0.21
D 3	-	-
D 4	0.32	0.15
D 5	0.20	0.27
D 6	-	14.21
D 7	0.55	0.18

## Data Availability

The data presented in this study are available on request from the corresponding author.

## Supplementary Materials

The following are available online at https://www.mdpi.com/article/10.3390/s21092948/s1, Figure S1: Images from an optical microscope presenting the morphology of films (mag ×1000) produced by spraying and the depth profiles presenting the roughness of the layers (mag ×1000): (a_1_,a_2_)—UD 1 dispersion; (b_1_,b_2_)—UD 2 dispersion, (c_1_,c_2_)—UD 3 dispersion, (d_1_,d_2_)—UD 5 dispersion, (e_1_,e_2_)—UD 6 dispersion., Figure S2: Images from an optical microscope presenting the morphology of films (mag ×1000) produced by drop-casting and the depth profiles presenting the roughness of the layers (mag ×1000): (a_1_,a_2_)—UD 1 dispersion; (b_1_,b_2_)—UD 2 dispersion, (c_1_,c_2_)—UD 3 dispersion, (d_1_,d_2_)—UD 5 dispersion, (e_1_,e_2_)—UD 6 dispersion., Figure S3: The resistance response of the sensors deposited from: (a) UD 2 dispersion by spraying, (b) UD 2 dispersion by drop-casting, (c) UD 4 dispersion by spraying, (d) UD 4 dispersion by drop-casting, (e) UD 5 dispersion by drop-casting, (f) UD 7 dispersion by spraying, (g) UD 7 dispersion by drop-casting.
