# Supplementary material for: Chemosensitive Thin Films Active to Ammonia Vapours
Source: Sensors (Basel). 2021 Apr 22;21(9):2948. doi: 10.3390/s21092948 (PMC8122796; doi:10.3390/s21092948)
Supplement: Supplementary file 1 [file sensors-21-02948-s001.zip › sensors-1186004-supplementary.pdf]

sensors

Supplementary Materials

Chemosensitive Thin Films Active to Ammonia Vapours

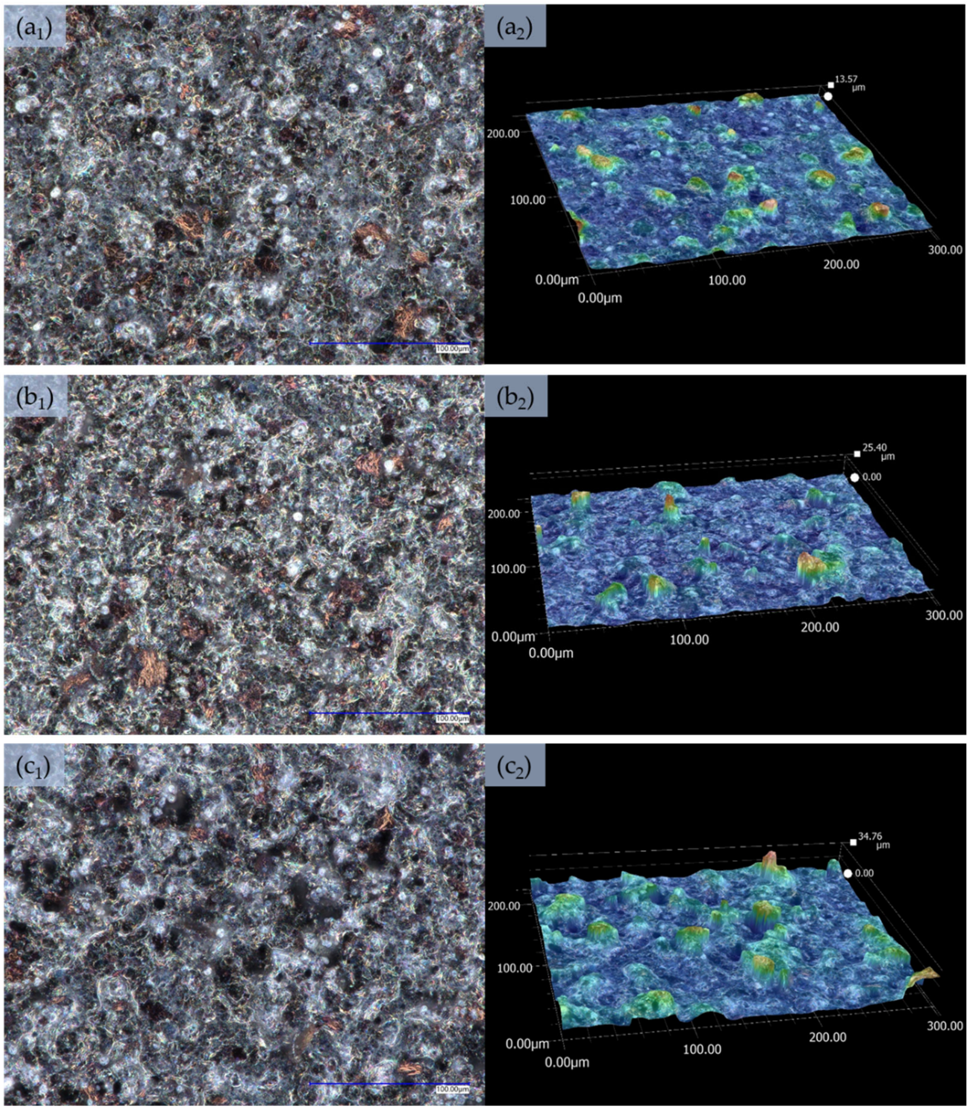

Citation: Brochocka, A.; Nowak, A.; Zajączkowska, H.; Sieradzka, M. Chemosensitive Thin Films Active to Ammonia Vapours. *Sensors* **2021**, *21*, 2948. https://doi.org/10.3390/s21092948

Academic Editor:

Received: 31 March 2021
Accepted: 19 April 2021
Published: date

Publisher's Note: MDPI stays neutral with regard to jurisdictional claims in published maps and institutional affiliations.

Copyright: © 2021 by the authors. Submitted for possible open access publication under the terms and conditions of the Creative Commons Attribution (CC BY) license (http://creativecommons.org/licenses/by/4.0/).Citation: Brochocka, A.; Nowak, A.; Zajączkowska, H.; Sieradzka, M.

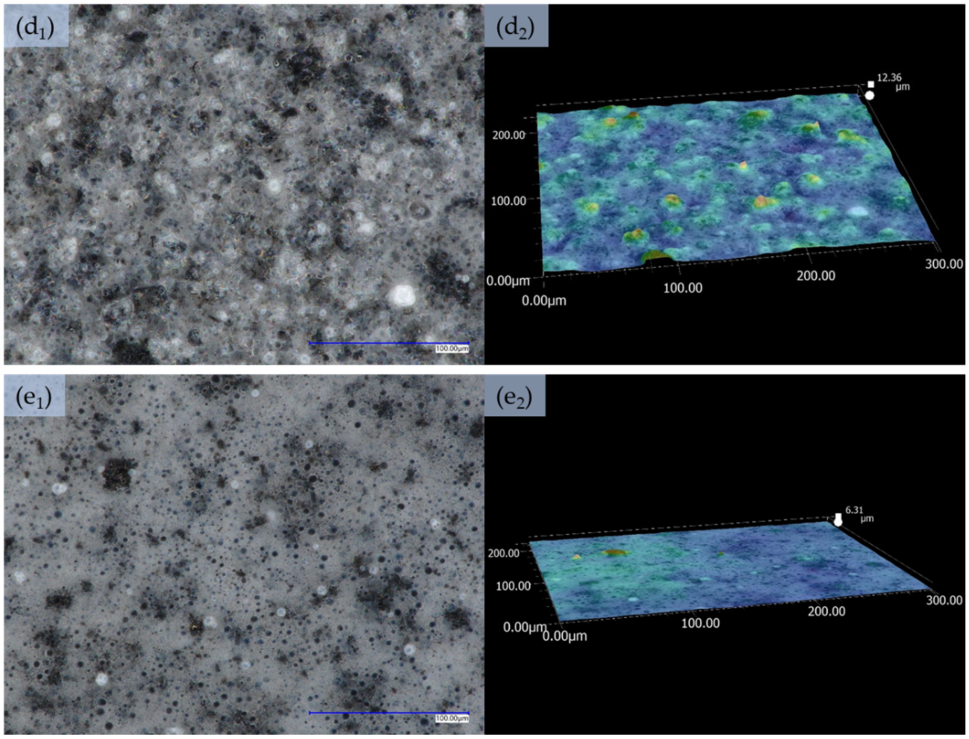

Figure 1. Images from an optical microscope presenting the morphology of films (magx1000) produced by spraying and the depth profiles presenting the roughness of the layers (magx1000): (**a1**), (**a2**) - UD 1 dispersion; (**b1**), (**b2**) - UD 2 dispersion, (**c1**), (**c2**) - UD 3 dispersion, (**d1**), (**d2**) - UD 5 dispersion, (**e1**), (**e2**) - UD 6 dispersion.

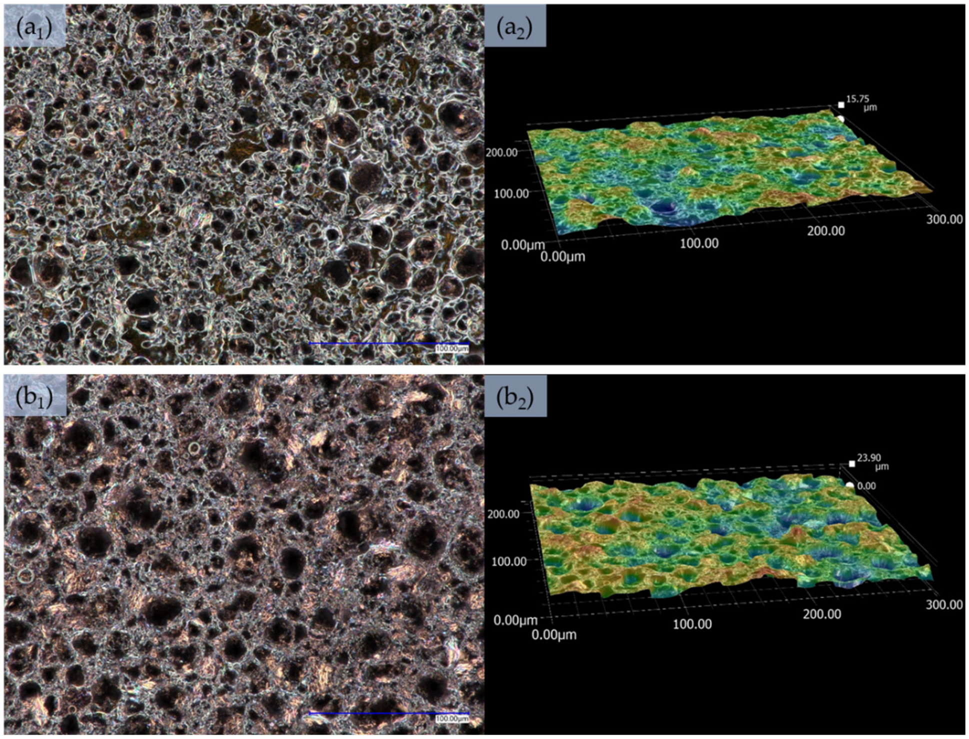

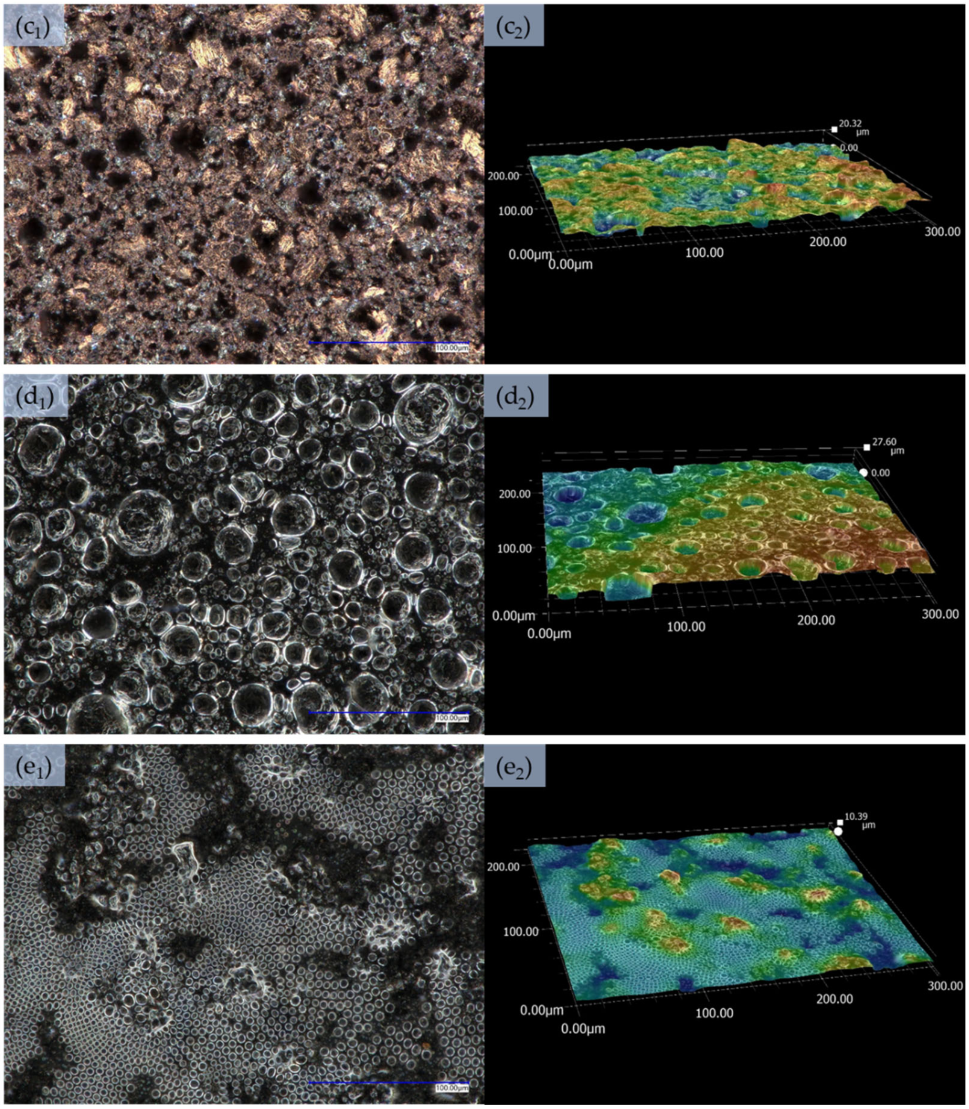

Figure 2. Images from an optical microscope presenting the morphology of films (magx1000) produced by drop-casting and the depth profiles presenting the roughness of the layers (magx1000): (**a1**), (**a2**) - UD 1 dispersion; (**b1**), (**b2**) - UD 2 dispersion, (**c1**), (**c2**) - UD 3 dispersion, (**d1**), (**d2**) - UD 5 dispersion, (**e1**), (**e2**) - UD 6 dispersion.

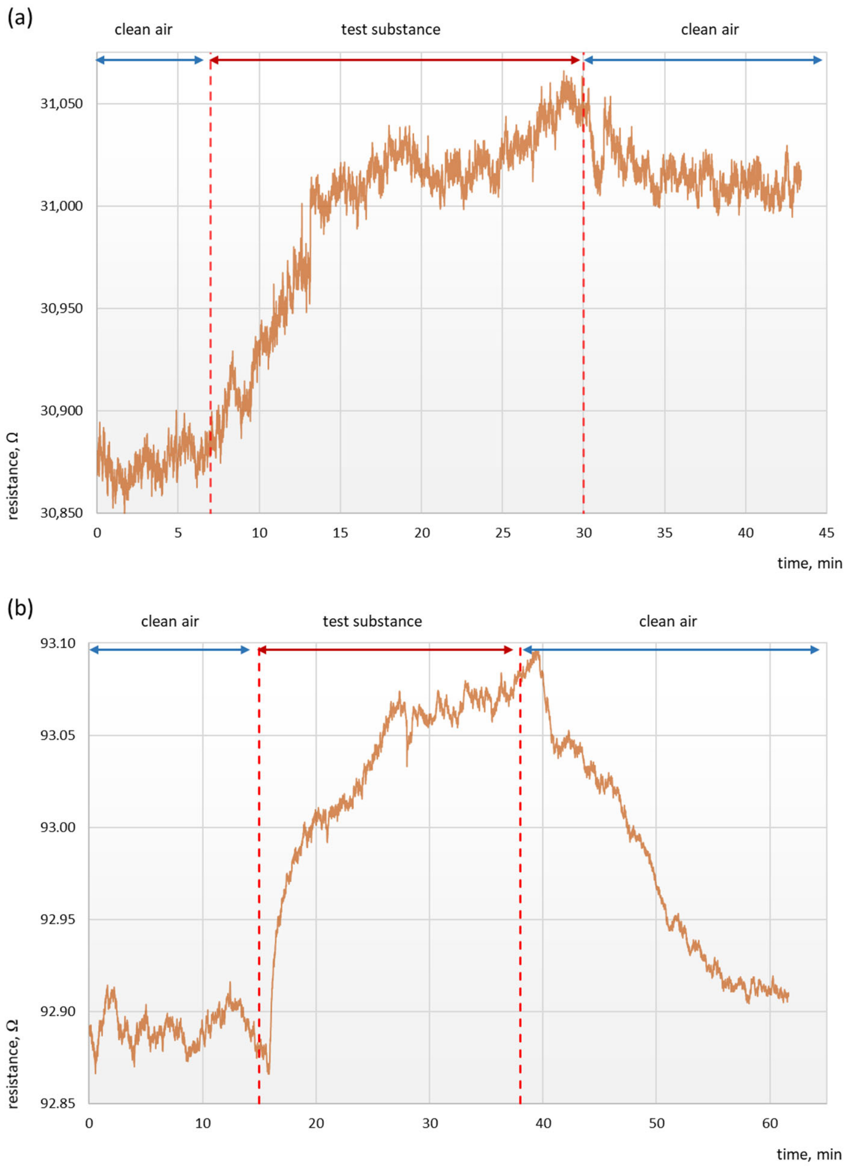

(a)
clean air
test substance
clean air
31,050
31,000
30,950
30,900
30,850
resistance, Ω
0
5
10
15
20
25
30
35
40
45
time, min
(b)
clean air
test substance
clean air
93.10
93.05
93.00
92.95
92.90
92.85
resistance, Ω
0
10
20
30
40
50
60
time, min

(c)

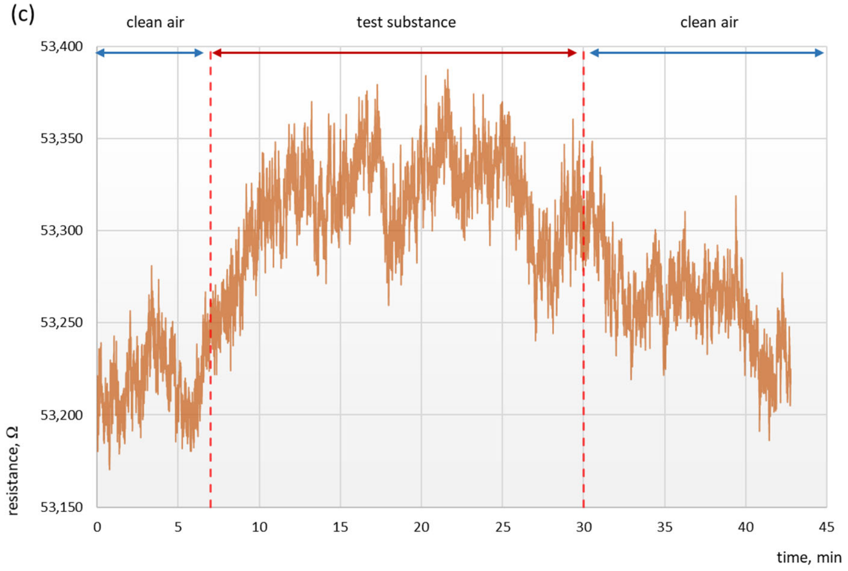
clean air
test substance
clean air
53,400
53,350
53,300
53,250
53,200
53,150
resistance, Ω
0
5
10
15
20
25
30
35
40
45
time, min

(d)

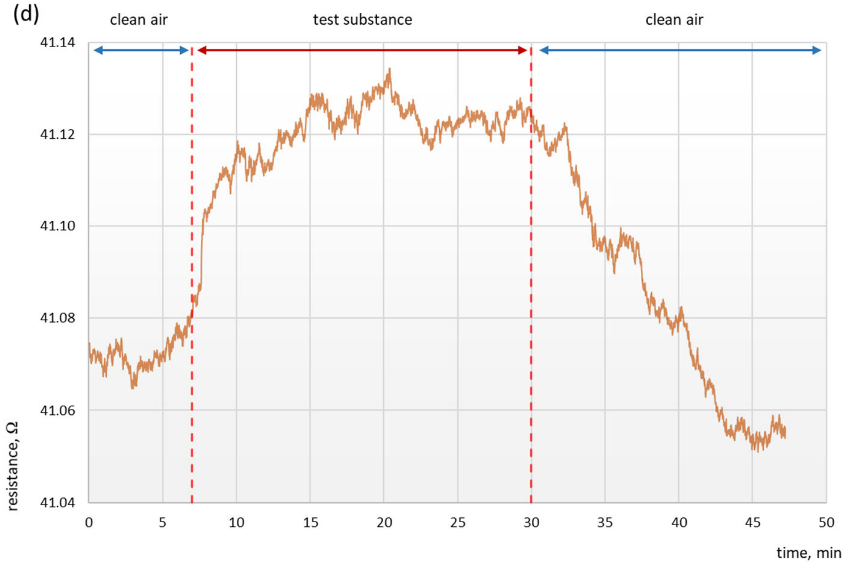
clean air
test substance
clean air
41.14
41.12
41.10
41.08
41.06
41.04
resistance, Ω
0
5
10
15
20
25
30
35
40
45
50
time, min

(e)

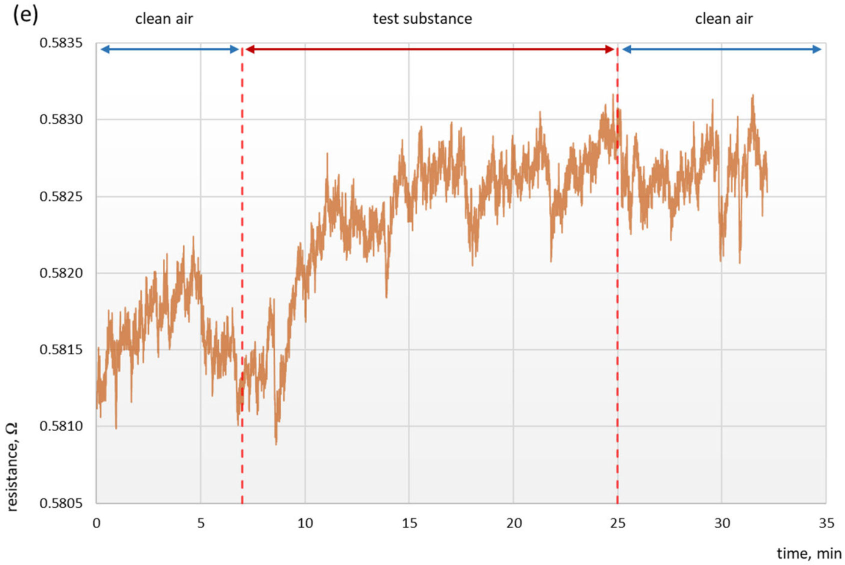
clean air
test substance
clean air
0.5835
0.5830
0.5825
0.5820
0.5815
0.5810
0.5805
resistance, Ω
0
5
10
15
20
25
30
35
time, min

(f)

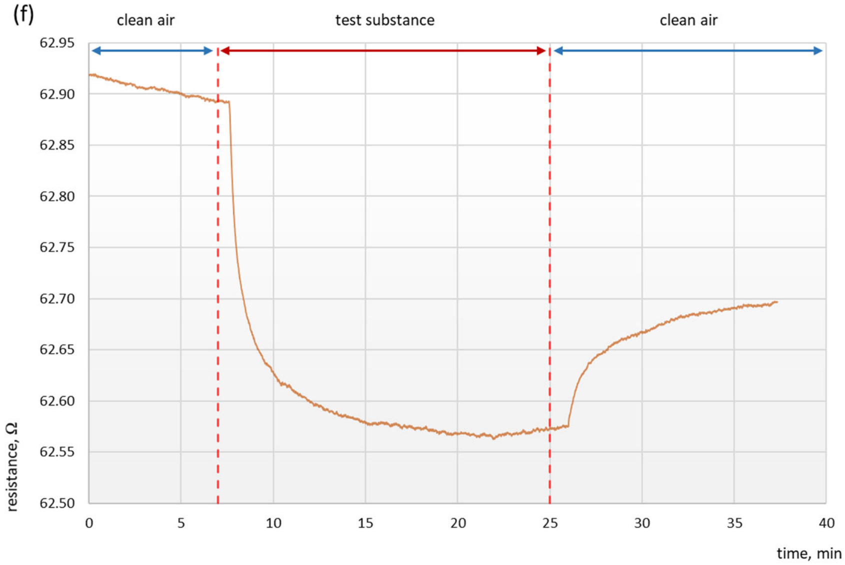
clean air
test substance
clean air
62.95
62.90
62.85
62.80
62.75
62.70
62.65
62.60
62.55
62.50
resistance, Ω
0
5
10
15
20
25
30
35
40
time, min

(g)

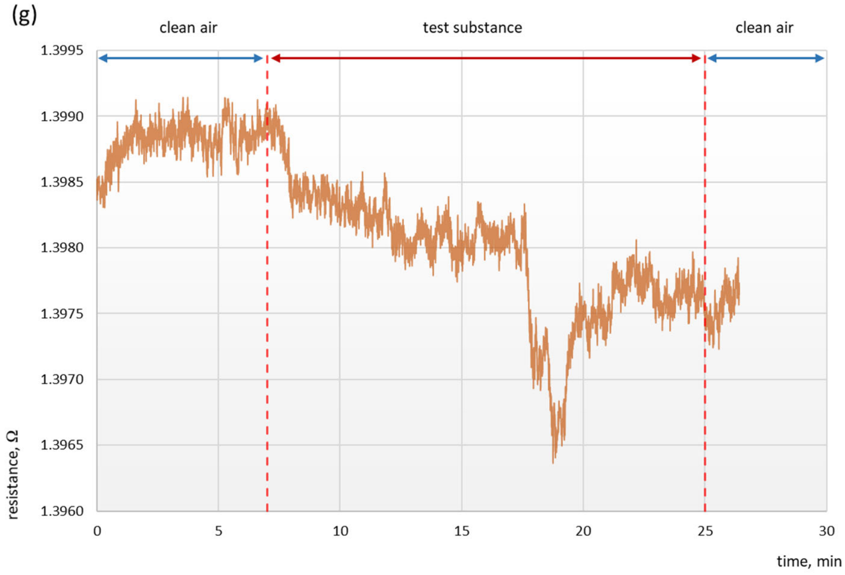
clean air
test substance
clean air
1.3995
1.3990
1.3985
1.3980
1.3975
1.3970
1.3965
1.3960
resistance, Ω
0
5
10
15
20
25
30
time, min

Figure 3. The resistance response of the sensors deposited from: (**a**) UD 2 dispersion by spraying, (**b**) UD 2 dispersion by drop-casting, (**c**) UD 4 dispersion by spraying, (**d**) UD 4 dispersion by drop-casting, (**e**) UD 5 dispersion by drop-casting, (**f**) UD 7 dispersion by spraying, (**g**) UD 7 dispersion by drop-casting.
